# Supplementary material for: MetaRibo-Seq measures translation in microbiomes
Source: Nat Commun. 2020 Jun 29;11:3268. doi: 10.1038/s41467-020-17081-z (PMC7324362; doi:10.1038/s41467-020-17081-z)
Supplement: Supplementary file 10 — Supplementary Data 7 [file 41467_2020_17081_MOESM10_ESM.zip › File2/Confidence_High_Taxonomy/58437_out.krona.html]

Javascript must be enabled to view this page.

members
magnitude
magnitudeUnassigned
count
unassigned
taxon
rank

58437\_out

9

2
superkingdom
9

phylum
1239
9

class
909932
9

9
order
1843489

9
family
31977

9
29465
genus


SRS012969\_contig\_number\_36546SRS014459\_contig\_number\_40516SRS015065\_contig\_number\_32179SRS015663\_contig\_number\_37150SRS051031\_contig\_number\_34349SRS065397\_contig\_number\_695SRS104693\_contig\_number\_12990SRS104975\_contig\_number\_915SRS146813\_contig\_number\_20972
1262980
species
9
